# Psychometric evaluation and wording effects on the Chinese version of the parent-proxy Kid-KINDL

**DOI:** 10.1186/s12955-016-0526-3

**Published:** 2016-09-05

**Authors:** Chih-Ting Lee, Chung-Ying Lin, Meng-Che Tsai, Carol Strong, Yi-Ching Lin

**Affiliations:** 1 Department of Family Medicine, National Cheng Kung University Hospital, College of Medicine, National Cheng Kung University, Tainan, Taiwan; 2 Institute of Physical Education, Health and Leisure Studies, College of Management, National Cheng Kung University, Tainan, Taiwan; 3 Department of Rehabilitation Sciences, Faculty of Health and Social Sciences, Hong Kong Polytechnic University, 11 Yuk Choi Road, Hung Hom, Kowloon, Hong Kong; 4 Department of Pediatrics, National Cheng Kung University Hospital, College of Medicine, National Cheng Kung University, 1 University Road, Tainan, 70101 Taiwan; 5 Department of Public Health, National Cheng Kung University Hospital, College of Medicine, National Cheng Kung University, Tainan, Taiwan; 6 Institute of Health Behaviors and Community Sciences, College of Public Health, National Taiwan University, Taipei, Taiwan

**Keywords:** Children, Confirmatory factor analysis, Kid-KINDL, Parent proxy, Wording effect

## Abstract

**Background:**

The pediatric quality of life (QoL) questionnaire, the child-rated Kid-KINDL, has wording effects. However, no studies have examined for its parallel questionnaire, the parent-proxy Kid-KINDL. This study aimed to examine the psychometric properties and wording effects of the parent-proxy Kid-KINDL.

**Methods:**

Parents with 8- to 12-year-old children (*n* = 247) completed the parent-proxy Kid-KINDL, 83 of them completed it again 7–14 days later, and 241 of their children completed the child-rated Kid-KINDL. Internal consistency was examined using Cronbach’s α; test-retest reliability and concurrent validity, using Pearson correlation coefficients (*r*); construct validity and wording effects, using confirmatory factor analyses (CFAs).

**Results:**

The internal consistency of the parent-proxy Kid-KINDL total score was acceptable (α = .86). Test-retest reliability (*r* = .33–.60) and concurrent validity (*r* = .27–.42) were acceptable or nearly acceptable for all subscales and the total score. The CFA models simultaneously accounting for QoL traits and wording effects had satisfactory fit indices, and outperformed the model accounting only for QoL traits. However, four subscales had unsatisfactory internal consistency, which might be attributable to wording effects.

**Conclusion:**

When children are unable to complete a QoL questionnaire, the parent-proxy Kid-KINDL can substitute with all due cautions to wording effects and inconsistent reliability among different raters.

## Background

Health-related quality of life (QoL) is an important overall health indicator for healthcare professionals who make clinical decisions, and many QoL instruments have been well developed [1, 2]. Some QoL instruments for children [1] use both a child self-report and a parent-proxy report, thereby providing healthcare professionals additional information about the child’s QoL. Despite being a secondary outcome measure for healthcare professionals, a parent-proxy report can be the primary outcome measure when the child is unable to make a self-assessment, for example, when the child has severe mental retardation [3, 4]. Therefore, the parent-proxy report is also an important instrument for making clinical decisions.

Translations of the child self-report of the Kid-KINDL, one commonly used QoL instrument, have been examined for psychometric properties in many languages [5–10]. According to the findings on the Chinese version in Taiwan [10], validating the child-reported Kid-KINDL involved assessing a wording concept in addition to the QoL concept. Positively worded and negatively worded items have different effects on the child-reported Kid-KINDL, and the Kid-KINDL showed substantially improved construct validity when the wording effect was considered.

The use of negatively worded items together with positively worded items is not consistently applied across different QoL instruments [1–5]. Some argue for combined use of negatively and positively worded items, because they can reduce or eliminate acquiescence bias, and ceiling or floor effects resulting from all “yes” or all “no” answers [11–13]. Despite these potential advantages, negatively worded items can confuse respondents because of increasing difficulty in interpreting items. There is a rising concern about their harmful effects on the covariance structure of the scale. For example, some studies found that three negatively worded items of the World Health Organization QoL questionnaire resulted in unsatisfactory item properties [14–16], and thus suggested deleting these items. As such, it is important to investigate whether there is wording effect in the parent-proxy Kid-KINDL, because the scale consists of both negatively and positively worded items. We also need to determine whether the threat of wording effects on construct validity, if it exists, can be minimized or controlled through statistical methods; which indicates that the construct validity of the scale is satisfactory under examination. As long as this parent-proxy scale is valid and reliable, healthcare providers may have more confidence to use it.

However, the parent-proxy Kid-KINDL had not been translated into Chinese, and its psychometric properties had not been evaluated for Taiwan’s population. Therefore, this study examined the psychometric properties and wording effects on our Chinese translation of the parent-proxy Kid-KINDL. Also, we hypothesized that this wording effect also existed in the parent-proxy Kid-KINDL because it contains parallel items.

## Methods

### Participants

Parents with 3rd- to 6th-grade children (8–12 years old) in southern Taiwan were the target sample. After signing informed consents, 247 parents filled out the parent-proxy Kid-KINDL, and 241 of their children completed the child-reported Kid-KINDL. After 7 to 14 days of the first test, 83 parents completed the parent-proxy Kid-KINDL again. All the children completed the child-reported Kid-KINDL in group (each group consisted of 10 to 15 children completed at the same time) under the supervision of one author (C-Y Lin) and one of the children’s teachers. In addition, the children took the parent-proxy Kid-KINDL and a background information sheet for their parents to complete at home. The 83 test-retest parent-proxies were also completed by the parents at home. The Institutional Review Board of National Cheng Kung University Hospital approved the study (IRB no: ER-98-0256), and all the participants handed in a written informed consent.

### Instruments

The parent-proxy Kid-KINDL was used to evaluate the children’s QoL, ranging from 1 to 5, with 1 meaning “*always*” and 5 meaning “*never*” for positively worded items, and 1 meaning “*never*” and 5 meaning “*always*” for negatively worded items. Six subscale scores (physical well-being, emotional well-being, self-esteem, family, friends, and school; each with 4 items) and one total score (24 items) can be computed and transformed into a 0–100 scale using the developers’ instructions [17]. A higher score stands for a better QoL. The parent-proxy Kid-KINDL was translated into Chinese for Taiwanese children using forward translation (by two independent Taiwanese translators), reconciliation (by two forward-translation translators and the second author), and backward translation (by one German translator). In addition, the backward-translation version was examined by the developers, and the final Chinese version was reworded by the second author until the developers accepted it. The child self-rated Kid-KINDL with items parallel to the parent-proxy Kid-KINDL has satisfactory psychometric properties [10], and was used as a criterion for the parent-proxy Kid-KINDL.

### Data analysis

Floor and ceiling effects were the responses at 0/100 divided by all responses (*n* = 247). We used Cronbach’s α coefficients to assess internal consistency; Pearson correlation coefficients (*r*) to assess test-retest reliability (between the first-time and second-time parent-proxy) and concurrent validity (between the parent-rated and child-rated Kid-KINDL) for each comparable subscale. Cronbach’s α > .7 suggests acceptable reliability [18], and an *r* > .3 suggests acceptable test-retest reliability and concurrent validity [19, 20]. The construct validity of the Kid-KINDL was examined using multitrait-multimethod (MTMM)-designed confirmatory factor analysis (CFA) with six competing models. We used seven models, six of which were the same models used in another study [10]; the seventh was a general model. One item (Fr4 “*felt different from other children*”) was eliminated for all CFAs based on the suggestion of previous studies [21–23]; the reasons of the elimination include (1) the uncertainty of the item concept belongs to positive wording [22, 23] or negative wording [17], and (2) the unsatisfactory internal consistency shown in previous study when including the item (α = 0.533 for the friend subscale using all items; α = 0.765 after deleting the item Fr4) [10]. Our current results also showed that the friend subscale had higher internal consistency when deleting the item (α = 0.79) than including the item (α = 0.63).

**Fig. 1:**
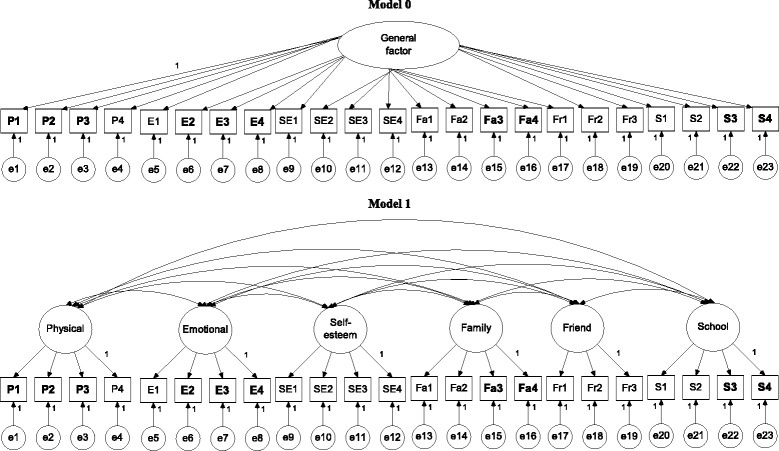
Model 1 is a 6-QoL-factor (physical, emotional, self-esteem, family, friend, and school) model; arrows without an assigned value were freely estimated; negatively worded items are in **bold**

**Fig. 2:**
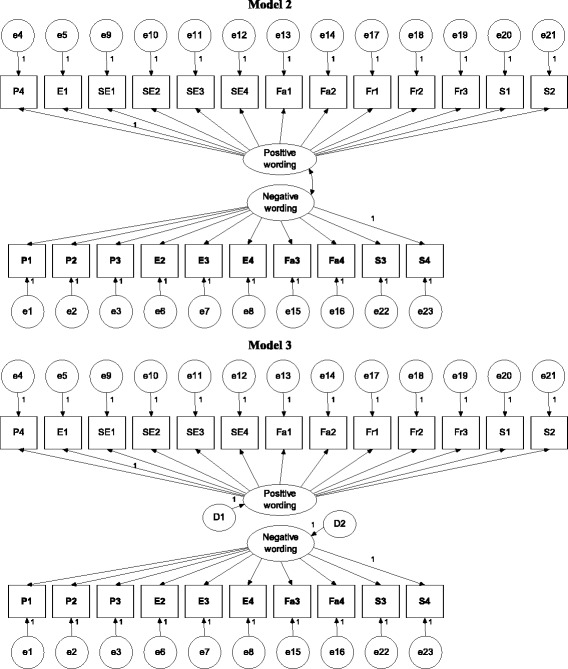
Models 2 and 3 are a 2-oblique-wording-factor (positive and negative wording) and a 2-orthogonal-wording-factor model, respectively; arrows without an assigned value were freely estimated; negatively worded items are in **bold**

**Fig. 3:**
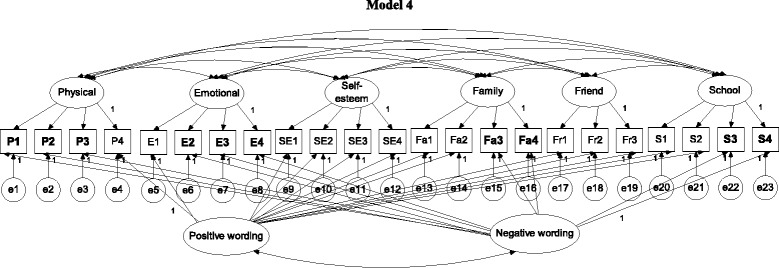
Model 4 is a correlated traits (QoL factors) and correlated methods (wording factors) model (CTCM model); arrows without an assigned value were freely estimated; negatively worded items are in **bold**

**Fig. 4:**
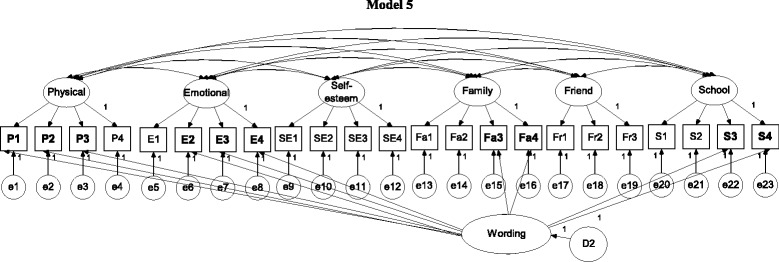
Model 5 is a correlated traits and one-wording-factor model (CTC [M − 1] model); arrows without an assigned value were freely estimated; negatively worded items are in **bold**

**Fig. 5:**
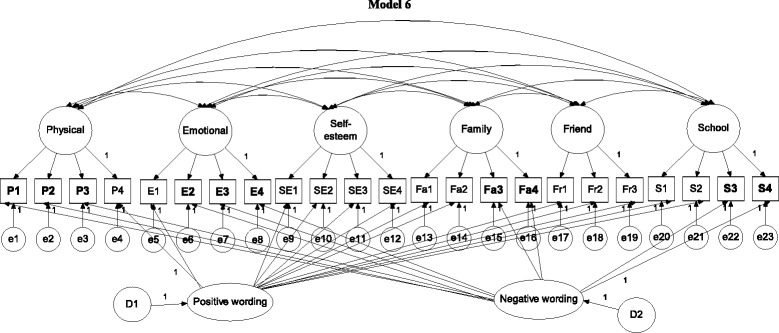
Model 6 is a correlated traits and uncorrelated methods model (CTUM model); arrows without an assigned value were freely estimated; negatively worded items are in **bold**

In brief, Model 0 (Fig. 1) was a one-general-factor model with all items loaded on the general factor; Model 1 (Fig. 1) was a six-QoL-factor model; Models 2 and 3 (Fig. 2) were two wording-factor models with oblique and orthogonal wording factors, respectively; Model 4 (Fig. 3) was a correlated traits (i.e., QoL factors) and correlated methods (i.e., wording factors) model (CTCM); Model 5 (Fig. 3) was a correlated traits and one-wording factor model (CTC [M − 1]); Model 6 (Fig. 4) was a correlated traits and uncorrelated methods model (CTUM). Arrows without any assigned value on Figs. 1, 2, 3, 4 and 5 were freely estimated for their coefficients, while those with a value of 1 had their loadings fixed as 1. In addition, no parameters were constrained to be equal.

If Model 1 outperforms Model 0, the result supports the multidimensionality (i.e., 6-factor structure) of the parent-proxy Kid-KINDL. If Models 2 and 3 outperform Model 0, the result supports the existence of wording effects in the parent-proxy Kid-KINDL. If Models 4 to 6 outperform Models 1 to 3, the results indicate that the wording effects and multidimensionality simultaneously exist in the parent-proxy Kid-KINDL. Also, the construct validity of the parent-proxy Kid-KINDL can be supported as a 6-factor framework.

Acceptable data-model fit indices in CFA models include a non-significant *χ*^2^; *χ*^2^/*df* < 2, goodness of fit index (GFI), Tucker-Lewis index (TLI), comparative fit index (CFI), and incremental fit index (IFI) > .9; and a root mean square error of approximation (RMSEA) and standardized root mean square residual (SRMR) < .08 [24, 25]. Given the high power of the *χ*^2^ test in a large sample, even a minor misfit of a model can result in its rejection; therefore, the other indices were used instead [26, 27]. In addition, we used 4 CFA models (Models 1, 4, 5, and 6) to test convergent and discriminant validity: high factor loadings on each Kid-KINDL subscale suggested satisfactory convergent validity; low correlation coefficients among different subscales indicated good discriminant validity [28]. However, because the acceptable factor loadings (for convergent validity) and correlation coefficients (for discriminant validity) may be varied among different fields, we proposed the cutoff of .3 for factor loadings and .7 for correlation coefficients [29] in this study. In other words, a factor loading < .3 indicates that an item is not well fit in its subscale; a correlation coefficient > .7 between two subscales suggests that the two subscales are not discriminant. Additionally, the 7 CFA models competed using a *χ*^2^ difference test to examine both trait and wording effects [28, 30]. Specifically, a significant *χ*^2^ difference test between Models 1 and 0 suggests discriminant trait effects, between Models 2–3 vs. 0 suggests discriminant wording effects, and between Models 4–6 and 1 suggests that both trait and method effects exist. SPSS 16.0 (SPSS Inc., Chicago, IL, USA) and AMOS 7.0 (SPSS Inc.) were used for the analyses.

## Results

Of the 247 participants, 57 were fathers, 178 mothers, 9 others, and 3 anonymous. The mean ± SD subscale scores they gave for their children’s QoL were 78.80 ± 16.42 (physical), 80.85 ± 13.42 (emotional), 71.15 ± 19.45 (self-esteem), 76.59 ± 14.65 (family), 76.82 ± 15.51 (friends), and 69.33 ± 16.90 (school). In addition, the mean total score was 75.59 ± 10.98. No floor effects were found in any subscales or the total score (0–1.2 %). Minimal ceiling effects were found in the subscales (5.3–15 %) but not in the total scores (0 %). We additionally compared the scores between child-rated and parent-proxy Kid-KINDL using a total of 241 child–parent pairs. Our results showed that parents tended to rate a higher score than did their children, particularly in total score and subscale scores of physical, self-esteem, friend, and school (Table 1). After removing negatively worded items, parents still rated a higher score than did their children. The significant differences were shown in total score and subscale scores of physical, self-esteem, and family (Table 1). Whether we decide to remove or retain negatively worded items from the scale should not change the score on the self-esteem subscale, because the subscale items are all originally positively worded. Thus, the significant differences between child-rated and parent-proxy Kid-KINDL were slightly different between analyzing all items and analyzing positively items only.

**Table 1:** Score comparisons between child-rated and parent-proxy Kid-KINDL (*n* = 241)

	Child-rated	Parent-proxy	*t*-value (*p*-value)
	Mean ± SD	Mean ± SD	
Retaining all negatively worded items			
Physical	75.86 ± 18.70	79.15 ± 16.29	2.41 (0.017)
Emotional	80.26 ± 17.85	81.09 ± 13.42	0.66 (0.51)
Self-esteem^a^	58.27 ± 27.79	71.40 ± 19.54	6.70 (<0.001)
Family	75.86 ± 17.91	76.64 ± 14.76	0.66 (0.51)
Friend	68.36 ± 18.94	77.23 ± 15.35	7.37 (<0.001)
School	63.54 ± 21.50	69.71 ± 16.77	4.49 (<0.001)
Total	70.36 ± 14.40	75.87 ± 10.92	5.99 (<0.001)
Removing all negatively worded items			
Physical	68.93 ± 29.21	80.16 ± 21.47	5.22 (<0.001)
Emotional	80.82 ± 23.71	84.02 ± 15.03	1.96 (0.051)
Self-esteem^a^	58.27 ± 27.79	71.40 ± 19.54	6.70 (<0.001)
Family	75.77 ± 20.15	80.68 ± 14.81	3.94 (<0.001)
Friend	85.90 ± 19.38	87.75 ± 14.55	1.38 (0.17)
School	76.02 ± 20.71	77.42 ± 17.36	1.01 (0.32)
Total	74.16 ± 16.91	80.35 ± 11.81	5.73 (<0.001)

^a^All items in self-esteem subscale are positively worded; thus, the scores were the same across retaining all negatively worded items and removing all negatively worded items

The internal consistency was acceptable for the total score and for the physical and self-esteem subscales. However, the others did not reach the.7 recommendation (α = .59–.64). For test-retest reliability, the correlation coefficients were adequate for all subscales and the total score. All subscales but the physical (*r* = .27), emotional (*r* = .25), and self-esteem (*r* = .21) had acceptable concurrent validity (Table 2).

**Table 2:** Internal consistency, test-retest reliability, and concurrent validity of the parent-proxy Kid-KINDL

		Cronbach’s α	*r* for test-retest	*r* for concurrent validity^a^
Subscales	No. of items	(*n* = 247)	(*n* = 83)	(*n* = 241)
Physical	4	.71	.56	.27
Emotional	4	.63	.63	.25
Self-esteem	4	.83	.33	.21
Family	4	.64	.43	.38
Friend	4	.63	.38	.42
School	4	.59	.54	.40
Total	24	.86	.60	.39

^a^Criterion for concurrent validity is the child-reported Kid-KINDL

Models 4 to 6 had better data-model fits than did Models 1 to 3. Except for the RMSEA (.07) in Model 1, none of the fit indices were acceptable in Models 1 to 3 (Table 3). In addition, all fit indices were acceptable in Models 4 to 6, except for the GFI (.89) in Model 5. The *χ*^2^ difference tests for Models 4–6 vs. 1 (Model 4 vs. 1: ∆*χ*^2^ [∆*df=24*] = 191.56; Model 5 vs. 1: ∆*χ*^2^ [∆*df=10*] = 126.15; Model 6 vs. 1: ∆*χ*^2^ [∆*df=23*] = 190.46; All *P*s < .0001) corresponded to the fit indices, which indicated existing wording effects. The physical subscale had a low correlation with the other subscales (*r* < .3), except for the emotional subscale (*r* = .31–.55). However, the emotional, self-esteem, friends, family, and school subscales were moderately correlated with each other (Table 4). Nevertheless, the correlations among the six subscales were not > .7, which indicated acceptable discriminant validity of the Kid-KINDL. Moreover, *χ*^2^ difference tests showed that Models 1 to 3 were significantly better than was Model 0 (Models 1 vs. 0: ∆*χ*^2^ [∆*df=16*] = 687.37; Models 2 vs. 0: ∆*χ*^2^ [∆*df=2*] = 379.61; Models 3 vs. 0: ∆*χ*^2^ [∆*df=1*] = 371.42; All *P*s < .0001), which indicated that both QoL traits and wording effects were discriminant. In addition, the correlation between the two methods was extremely weak (*r* = .05) and nonsignificant in Model 4.

**Table 3:** Goodness-of-fit indices for parent-proxy Kid-KINDL^a^ (*n* = 247)

Variables	Model 0	Model 1	Model 2	Model 3	Model 4	Model 5	Model 6
*χ* ^2^	1159.51*	472.14*	779.90*	788.09*	280.58*	345.99*	281.68*
*df*	231	215	229	230	191	205	192
*χ* ^2^/*df*	5.02	2.20	3.41	3.43	1.47	1.67	1.47
GFI	.69	.85	.78	.78	.91	.89	.91
TLI	.51	.86	.74	.71	.94	.92	.94
CFI	.56	.88	.74	.73	.96	.93	.96
IFI	.56	.88	.74	.74	.96	.93	.96
RMSEA	.13	.07	.10	.10	.04	.05	.04
SRMR	.13	.09	.10	.11	.05	.07	.05

Model 0 is a 1-general-factor model

Model 1 is a 6-QoL-factor (physical, emotional, self-esteem, family, friend, and school) model

Model 2 is a 2-oblique-wording-factor (positive and negative wording) model

Model 3 is a 2-orthogonal-wording-factor (positive and negative wording) model

Model 4 is a correlated traits (QoL factors) and correlated methods (wording factors) model (CTCM model)

Model 5 is a correlated traits and one-wording-factor model (CTC [M − 1] model)

Model 6 is a correlated traits and uncorrelated methods model (CTUM model)

*GFI* goodness-of-fit Index, *TLI* Tucker-Lewis Index, *CFI* comparative fit index, *IFI* incremental fit index, *RMSEA* root mean square error of approximation, *SRMR* standardized root mean square residual

*P < .05

^a^Item Fr4 (felt different from other children) was eliminated in all CFA models based on the suggestion of previous studies (Helseth & Lund [21]; Lin et al. [10]; Lee et al. [22]; Wee et al. [23])

**Table 4:** Correlations between QoL factors in models^a^ (*n* = 247)

Subscales	Physical	Emotional	Self-esteem	Family	Friends
Model 1 (QoL model)/Model 4 (CTCM) ^b^					
Emotional	.55/.48				
Self-esteem	.10/.25	.41/.47			
Family	.24/.29	.51/.49	.69/.61		
Friends	.04/.12	.40/.42	.68/.35	.57/.38	
School	.14/.16	.38/.32	.62/.40	.51/.33	.57/.33
Model 5 (CTC [M − 1])/Model 6 (CTUM)					
Emotional	.31/.48				
Self-esteem	.20/.25	.67/.50			
Family	.27/.30	.64/.51	.69/.63		
Friends	.13/.13	.63/.44	.68/.38	.57/.40	
School	.18/.17	.51/.35	.63/.44	.51/.37	.57/.36

Model 1 is a 6-QoL-factor (physical, emotional, self-esteem, family, friend, and school) model

Model 4 is a correlated traits (QoL factors) and correlated methods (wording factors) model (CTCM model)

Model 5 is a correlated traits and one-wording-factor model (CTC [M − 1] model)

Model 6 is a correlated traits and uncorrelated methods model (CTUM model)

^a^Item Fr4 (felt different from other children) was eliminated in all CFA models based on the suggestion of previous studies (Helseth & Lund [21]; Lin et al. [10]; Lee et al. [22]; Wee et al. [23])

^b^The correlation coefficient between the two methods (positive wording vs. negative wording) was.05

Convergent validity results showed that most items fit well in their assigned subscales in the QoL-related models (i.e., Models 1, 4, 5, and 6). However, six items (Items P4 “*strong and full of energy*”, SE1 “*proud of myself*”, SE4 “*had lots of good ideas*”, Fa4 “*stopped from doing certain things*”, S3 “*worried about my future*”, and S4 “*was afraid of bad marks or grades*”) did not reach the suggested .3 cutoff in some or all QoL-related models (Table 5).

**Table 5:** Standardized factor loadings in confirmatory factor analysis (CFA) models (*n* = 247)

Domain	Model #		Domain	Model #	
Item #	Models 1/4	Models 5/6	Item #	Models 1/4	Models 5/6
Physical			Family		
*P1*	.84/.83	.73/.83	Fa1	.79/.68	.78/.69
*P2*	.82/.78	.68/.78	Fa2	.87/.75	.88/.76
*P3*	.73/.69	.62/.69	*Fa3*	.41/.42	.40/.43
P4	.24/.27	.33/.27	*Fa4*	.23/.28	.21/.28
Emotional			Friends^a^		
E1	.51/.44	.74/.46	Fr1	.56/.40	.56/.41
*E2*	.38/.30	.34/.33	Fr2	.81/.48	.80/.49
*E3*	.67/.67	.42/.67	Fr3	.91/.86	.91/.86
*E4*	.67/.55	.39/.56			
Self-esteem			School		
SE1	.64/.23	.64/.27	S1	.83/.93	.82/.91
SE2	.81/.59	.81/.62	S2	.85/.57	.86/.61
SE3	.81/.45	.82/.48	*S3*	.14/.05	.15/.07
SE4	.70/.29	.70/.33	*S4*	.24/.22	.25/.24

Model 1 is a 6-QoL-factor (physical, emotional, self-esteem, family, friend, and school) model

Model 4 is a correlated traits (QoL factors) and correlated methods (wording factors) model (CTCM model)

Model 5 is a correlated traits and one-wording-factor model (CTC [M − 1] model)

Model 6 is a correlated traits and uncorrelated methods model (CTUM model)

Negatively worded items are in *italics*

^a^ Item Fr4 (*felt different from other children*) was eliminated in all CFA models based on the suggestion of other studies (Helseth & Lund [21]; Lin et al. [10]; Lee et al. [22]; Wee et al. [23])

## Discussion

Generally speaking, our results suggest practically acceptable reliability and validity for the Chinese version of the parent-proxy Kid-KINDL scores. The internal consistency in our study (α = .59–.86) corresponds to the previous data from Germany (α = .59–.86) [5], Norway (α = .67–.89) [6], and Serbia (α = .50–.85) [7]. Our findings are also comparable to the Chinese version of the child-reported Kid-KINDL (α = .52–.87) [10]. In addition, the four subscales with α < .7 in the current study had low values of subscale internal consistency that was also found in other studies [5, 7, 10]. One reason for the low internal consistency might be a small number of items (4 items) that were included in each subscale. Another reason might be that both positively and negatively worded items were concomitantly used in these subscales [10, 13]. Given that there was sound test-retest reliability for the total score, this suggested the stable reproducibility of the parent-proxy Kid-KINDL. Concurrent validity also shows that the parent-proxy Kid-KINDL score is comparable to the child-rated Kid-KINDL score. The clinical utility of the parent-proxy Kid-KINDL could be inferred when assessing child quality of life and care.

Construct validity as well as the wording effects were evaluated and supported by our six CFA models. Model 1, which considered no wording effects, showed that all fit indices were unacceptable. The fit indices of Models 4 to 6, which considered wording effects, were substantially better than those of Model 1. Specifically, the construct of parent-rated Kid-KINDL was established when accounting for wording effects, and indirectly supported that wording effects exist. The performance of these four models in the present study is comparable to the child-reported Kid-KINDL study [10], which also demonstrated the best model fit in Models 4 and 6. Therefore, we have extended the results of wording effects to the parent-proxy Kid-KINDL. Nevertheless, our finding which showed that the method effects of item wording were artifacts (i.e., one kind of error caused by response style) agreed with those of other studies on the Rosenberg self-esteem scale [31–33]. Although the artifacts might be invariant over time [33, 34], we propose that rewording these sentences so that they express clear concepts will solve this problem. The effect of artifacts on the parent-proxy Kid-KINDL should be reduced in the future refinement of the questionnaire.

Based on the results of factor loadings, six items did not fit quite well in their originally assigned subscales. Despite its unfulfilled criteria of model fit, we still could justify the factor loadings of items SE1 (*proud of myself*) and SE4 (*had lots of good ideas*) remaining in the self-esteem subscale, as they are proved acceptable in Models 1 and 4, and almost acceptable in Models 5 and 6. Further, we argued for some additional modifications for the other four items with the following considerations. Item P4 (*strong and full of energy*) reflects an overall physical condition and might be slightly out of the physical subscale because the other three items on this subscale (*felt ill*; *headache or stomachache*; *tired and sleepy*) measure mainly physical problems. Item Fa4 (*stopped from doing certain things*) might not directly measure the quality of family life because it somehow mixes with the concept of parental monitoring and social control behavior [35]. Item S3 (*worried about my future*) also could not be able to be confined within the school domain because *future* is related to many factors (e.g., friendship, self-esteem, and family context), and *worry* contains the concept of emotions. Likewise, item S4 (*was afraid of bad marks or grades*) combines the concepts of school and emotions.

This study has some limitations. First, we did not use an experimental design to tackle the effects of positively and negatively worded items in the Kid-KINDL. The best method to determine the wording effects is to compare two versions of questionnaires (the original and another with all negatively worded items) or three versions of questionnaires (the original, one with all negatively worded items, and one with all positively worded items). The comparisons will then provide us informative findings to explore the existence of the wording effects. However, we did not do so because Kid-KINDL is an established and standardized instrument across many countries, and we did not have the permission to revise the structure of the Kid-KINDL. Hence, future studies with an experimental design may be needed to elaborate the issues of wording effects for Kid-KINDL. Second, raters of different genders (e.g., father and mother) participated in this study, and they might rate their children’s QoL differently. Jozefiak et al. [6] reported that father-proxy and mother-proxy reports were only moderately correlated. Gender and other personal factors may explain the substantial disagreement among raters of different categories. Third, none of the parents who participated in this study had children being diagnosed with or under medical treatments for health problems. This would limit the generalizability of our findings to specific clinical conditions that are potentially related to the impaired QoL. Validation of these results in clinical samples of children would be needed to explore the disease-specific utility. Fourth, the use of MTMM-designed CFA models did not meet the basic requirements of at least 3 traits and 3 methods [28, 30, 36]. This is particularly because of the wording effects: there is no third method that can examine the wording effects when considering positive and negative wordings. Most studies on positive and negative wording effects using MTMM-designed CFA models [10, 33, 34, 37] also encountered the same problems as we did. Therefore, alternative approaches such as two methods [38, 39] or only two traits [40] have been proposed to tackle this problem. Our use of the two methods has shown the acceptability of the parent-proxy Kid-KINDL in terms of its psychometric properties.

Based on our findings, future studies may need to further investigate in the following topic: whether removing or retaining negatively worded items from the subscales affects the comparison of QoL scores across children and their parents. We assume that removing or retaining these items will impact the QoL results because children and parents may have different interpretations on the same negatively worded items. Our assumption can somewhat be supported by the results of Table 1, which demonstrates that disparity between child-rated and parent-proxy Kid-KINDL scores is subject to different analyses using all Kid-KINDL items or removing negatively worded items. Despite our finding, future research is needed to support our surmise. Specifically, because of variation in individual perceptions and interpretations, the measures of QoL will yield different results across child-rated and parent-proxy Kid-KINDL. Given this problem, differential item functioning analysis [41–43] can be carried out to better understand the impacts of negatively worded items in the QoL instrument.

## Conclusion

In sum, the present study validated that the Chinese version of the parent-proxy Kid-KINDL could be a feasible substitute for the child-rated Kid-KINDL in Taiwan. In addition, the wording effects were demonstrated in the parent-proxy Kid-KINDL, as shown in the child-rated Kid-KINDL. Therefore, we tentatively conclude that using the parent-proxy Kid-KINDL for children who are unable to answer the child-rated Kid-KINDL seems plausible in some clinical situations, where children are too young or too sick to self-report. Because the wording effects pertained to the parent-proxy Kid-KINDL, there might be inconsistency in measurement as different raters (e.g., fathers, mothers, and other relatives) gave their report. Thus, caution is required when using its scores as proxy for children’s self-report QoL, especially in some subscales. Future studies should be aimed at improving the psychometric quality of the parent-proxy Kid-KINDL and the clinical validation.

## Abbreviation

CFA: Confirmatory factor analysis
CFI: Comparative fit index
CTCM: Correlated trait and correlated method model
CTUM: Correlated trait and uncorrelated method model
GFI: Goodness of fit index
IFI: Incremental fit index
MTMM: Multitrait multimethod
QoL: Quality of life
RMSEA: Root mean square error of approximation
SRMR: Standardized root mean square residual
TLI: Tucker-Lewis index
